# Carryover effects of dietary arginine on productive performance and blood biochemistry in Japanese quail (*Coturnix japonica*)

**DOI:** 10.1016/j.vas.2026.100775

**Published:** 2026-07-19

**Authors:** Mequanint Gashew, Gebrehaweria K. Reda, Renáta Knop, Csaba Szabó, Ádám Z. Lendvai, Levente Czeglédi

**Affiliations:** a Department of Animal Science, Institute of Animal Science, Biotechnology and Natural Conservation, Faculty of Agricultural and Food Sciences and Environmental Management, University of Debrecen, Debrecen, 4032, Hungary; b Doctoral School of Animal Science, University of Debrecen, Debrecen, 4032, Hungary; c Department of Evolutionary Zoology and Human Biology, University of Debrecen, Debrecen, 4032, Hungary; d Department of Animal Nutrition and Physiology, Faculty of Agriculture and Food Sciences and Environmental Management, University of Debrecen, Debrecen, 4032, Hungary; e Department of Animal Science, Agriculture and Environmental Science College, Debre Tabor University, Debre Tabor, 272, Ethiopia

**Keywords:** Carryover, Arginine, Quail, Biochemistry

## Abstract

Early nutritional programming can affect later development in birds, yet the carryover effects of dietary arginine in Japanese quail remain poorly understood. Arginine is an essential amino acid for birds, as they cannot synthesise it endogenously, and it plays a vital role in growth and metabolism. This study aimed to evaluate the effect of dietary arginine restriction or supplementation on body mass change, reproduction, and plasma biochemistry in Japanese quail after the dietary treatment was discontinued for two consecutive weeks. Birds were fed either a low-arginine diet (25% below recommendations), a control diet (recommendations), or a high-arginine diet (25% above recommendations) at the early reproductive stage and adult stage and were then subsequently returned to a standard diet for two weeks. Overall, in comparison with the immediate effects of dietary arginine, no significant residual effects of dietary arginine were observed on body mass change, average daily body mass gain, feed conversion ratio, and egg production after the withdrawal period. However, age-dependent responses were observed, and adult birds showed a significant decrease in body mass change as compared to early reproductive stage birds. In addition, cholesterol showed age-related differences, whereas alanine aminotransferase (ALT/GPT) and aspartate aminotransferase (AST/GOT) showed both age- and treatment related difference with adult birds exhibiting lower concentrations than early-reproductive-stage birds. These findings suggest that early-life dietary arginine manipulation has a limited influence on growth and reproduction after withdrawal but may induce age-dependent changes in selected biochemical parameters in Japanese quail.

## Highlights

• Arginine had no clear persistent effect on body mass change or egg production.
• Early life arginine treatment influenced plasma biochemical parameters.
• Age influenced plasma biochemical parameters.

## 1 Introduction

Early-life nutritional experiences play a crucial role in shaping the phenotypic expression of vertebrates (Bruinjé et al., 2019; Shephard et al., 2024). Nutritional conditions during early development can induce carryover effects, which persist beyond the feeding period and influence subsequent physiological, hormonal, and metabolic processes (Hu et al., 2008; O’Connor et al., 2014). Such effects may alter growth trajectories, fitness, and life-history traits (Harrison et al., 2011; Ryan Norris & Marra, 2007). In animals, early nutritional conditions influence later performance, indicating persistent effects on developmental programming (da Silva et al., 2024, 2025). In nutrition, amino acids play an important role in regulating growth, metabolism, and physiological functions. In avian species, particularly in quails, the types and levels of amino acids affect blood biochemical parameters, reflecting their influence on metabolic and physiological status (Alagawany et al., 2014; Atakisi et al., 2009; Tomaszewska et al., 2025).

Among these nutrients, arginine is of particular interest as an essential amino acid for birds, as they are unable to synthesise it endogenously, and it is involved in protein synthesis, immune regulation, and metabolic processes (Hassan et al., 2021; Wang et al., 2014). Consequently, to fulfil their physiological requirements, birds rely on dietary arginine (Brugaletta et al., 2023). Thus, dietary arginine is a suitable nutritional factor for investigating whether a short-term intervention has a subsequent carryover effect on the physiology of quail after the treatment is discontinued. In Japanese quail (*Coturnix japonica*), arginine supplementation has been associated with improved growth rate, antioxidant status, and reproductive traits (Kalvandi et al., 2022; Kheiri & Landy, 2020; Sousa et al., 2022). Previous studies from broilers and quail suggest that early-life arginine nutrition exerts lasting effects on growth and metabolism (Al-Daraji & Salih, 2012; Deng et al., 2005). However, the mechanism underlying the long-term effects is unclear. Early nutritional programming affects metabolism, organ development and enzymatic activity, thereby affecting physiological function beyond the treatment period (Abou-Elnaga & Selim, 2018; Ivarsson et al., 2022; Noy & Uni, 2010). Consequently, little is known about whether temporary dietary arginine restriction or supplementation during early life produces persistent effects on growth, reproduction, and metabolic health in Japanese quail.

To assess the nutritional carryover effects of early-life dietary arginine, blood biochemical parameters provide valuable indicators of metabolic and physiological status (Agina et al., 2017; Scholtz et al., 2009; Zálešáková et al., 2025). Enzymes such as Alanine aminotransferase (ALT/GPT) and Aspartate aminotransferase (AST/GOT) reflect liver function and metabolic activity. In addition, glucose, triglycerides, albumin, total protein, and cholesterol show the nutritional status of protein and energy metabolism. Therefore, changes in these biochemical parameters may indicate alterations in metabolic processes due to early dietary arginine intervention. Although evidence in Japanese quail is limited, the effects of early nutrition programming shape the subsequent late-life histories of poultry species (Hollemans et al., 2018; Hussain et al., 2018). In our previous studies, dietary arginine restriction reduced growth, whereas supplementation enhanced egg production during the treatment period (Gashew et al., 2025, 2026a), suggesting the effect of arginine in physiological processes. We selected two age groups: the early reproductive stage and the adult phase of Japanese quail, as they have different physiological conditions and life histories. The early reproductive-stage birds are still growing and undergoing sexual maturation, whereas adult birds are sexually mature (Ibrahim et al., 2015; Taghipour-Shahbandi et al., 2024). Therefore, understanding whether the effects of dietary arginine are maintained beyond the period of treatment in two different age groups of quails is important to increase production efficiency. However, it is not clear whether such an effect is persistent after the birds resume normal feeding. Therefore, this study aimed to evaluate whether early-life dietary arginine restriction or supplementation has carryover effects on body mass change, average daily body mass gain, egg production, feed intake, feed conversion ratio (FCR), and plasma biochemical parameters in Japanese quail.

## 2 Materials and methods

### 2.1 Experimental animal management

The experiment was conducted in compliance with the EU Directive 2010/63/EU (Protocol No 5/2021/DEMAB) at the Animal House of the Institute of Animal Science, Biotechnology, and Nature Conservation, University of Debrecen, Hungary, using female Japanese quails (*Coturnix japonica*). During the nutritional treatment phase, grower birds from three to four weeks of age and adults from seven to eight weeks of age received diets for 14 days with 25% restriction or supplementation relative to the recommended levels (NRC, 1994) (Tables 1 and 2). For grower birds, the control, arginine-supplemented, and arginine-restricted diets contained 1.25%, 1.56%, and 0.94% arginine, respectively (Gashew et al., 2025). Likewise, for adult birds, the control, arginine-supplemented, and arginine-restricted diets contained 1.26%, 1.575%, and 0.945% arginine, respectively (Gashew et al., 2026a). Following the completion of the nutritional trial phase, each age group of birds received a control diet for 14 days (Table 2). This carryover trial was conducted to see whether the previous arginine restriction and supplementation have a persistent effect on the performance of quail during the early reproductive stage (5–6 weeks of age) and adult phase (9–10 weeks of age). Throughout the experiment, environmental conditions were kept constant at 24 ± 3 °C, with 60–75% relative humidity and a 12:12 h light-dark cycle during the grower phase and a 14:10 h light-dark cycle during the adult phase.

**Table 1:** Experimental timeline and treatment phases.

Experimental phase	Age of quail	Treatment	Trial period
Nutritional phase	3–4 weeks	25% restriction of arginine, Control, and 25% supplementation of arginine	14 days
Carryover phase	5–6 weeks	Control diet	14 days
Nutritional phase	7–8 weeks	25% restriction of arginine, Control, and 25% supplementation of arginine	14 days
Carryover phase	9–10 weeks	Control diet	14 days

**Table 2:** Feed composition (%) and calculated nutrient content of the experimental diets.

	Grower phase			Adult phase		
Ingredients	Treatments			Treatments		
	Control	Low arginine	High arginine	Control	Low arginine	High arginine
Corn	3.40	3.40	3.40	9.53	9.53	9.53
Wheat	30.00	30.00	30.00	20.00	20.00	20.00
Corn germ meal	39.19	39.19	39.19	34.28	34.28	34.28
Corn gluten meal	10.90	10.90	10.90	2.24	2.24	2.24
Soybean meal	-	-	-	12.81	12.81	12.81
Fishmeal	3.74	3.74	3.74	-	-	-
Sunflower oil	8.08	8.08	8.08	11.91	11.91	11.91
Limestone	1.40	1.40	1.40	5.93	5.93	5.93
MCP	0.42	0.42	0.42	1.01	1.01	1.01
L-Lys	0.77	0.77	0.77	0.38	0.38	0.38
DL-Met	0.06	0.06	0.06	0.15	0.15	0.15
L-Thr	0.28	0.28	0.28	0.11	0.11	0.11
L-Trp	0.06	0.06	0.06	0.02	0.02	0.02
L-Ile	0.20	0.20	0.20	-	-	-
L-Arg	0.39	0	0.78	0.39	0.00	0.78
Inert (kaolin)	0.39	0.78	0	0.39	0.78	0.00
Salt	0.22	0.22	0.22	0.35	0.35	0.35
Premix^a^	0.50	0.50	0.50	0.50	0.50	0.50
Nutrient content						
ME (MJ/kg)	12.13	12.13	12.13	12.13	12.13	12.13
Crude protein (%)	22.00	22.00	22.00	18.000	18.000	18.000
Lys (%)	1.30	1.30	1.30	1.000	1.000	1.000
Met (%)	0.50	0.50	0.50	0.450	0.450	0.450
Met+Cys (%)	-	-	-	0.781	0.781	0.781
Thr (%)	1.02	1.02	1.02	0.740	0.740	0.740
Trp (%)	0.22	0.22	0.22	0.190	0.190	0.190
Leu (%)	2.16	2.16	2.16	1.469	1.469	1.469
Ile (%)	0.98	0.98	0.98	0.668	0.668	0.668
Arg (%)	1.25	0.94	1.56	1.260	0.945	1.575
Leu/Ile	2.20	2.20	2.20	2.200	2.200	2.200
Ca (%)	0.80	0.80	0.80	2.500	2.500	2.500
Total Phosphorus (%)	0.56	0.56	0.56	0.599	0.599	0.599
Available Phosphorus (%)	0.30	0.30	0.30	0.350	0.350	0.350
Na (%)	0.15	0.15	0.15	0.150	0.150	0.150
DCAB, mEq/kg	-	-	-	96.7	96.7	96.7

a The premix provided the following per kilogram of complete diet: 5000 IU vitamin A, 1000 IU vitamin D3, 24.5 mg kg^-1^ vitamin E, 1 mg vitamin K3, 0.75 mg vitamin B1, 2.5 mg vitamin B2, 6 mg Ca-d-Pantothetane, 2 mg vitamin B6, 10 ug vitamin B12, 55 µg biotin, 12.5 mg niacin, 0.3 mg folic acid, 1500 mg choline chloride, 66 mg Zn, 9.6 mg Cu, 48.1 mg Fe, 66 mg Mn, 0.9 mg I, 0.21 mg Se, 60 µg Co. MCP: monocalcium phosphate; ME: metabolisable energy; DCAB: dietary cation-anion balance (electrolyte balance).

### 2.2 Experimental design and sampling

The experiment was carried out independently in two age groups of female Japanese quail: the early reproductive stage and adult phase. For each age group, birds were randomly assigned to one of the three dietary treatment groups (low arginine, control, and high arginine) with three replicate cages per treatment and six birds per replicate, for a total of 18 birds per treatment and 54 birds per age group. At the end of the 14-day experimental period, eight birds were randomly selected from each treatment (24 birds per age group) for organ mass measurements and plasma biochemical analyses. Quails were housed in identical cages (45 cm × 52 cm × 27 cm; length × width × height) with a stocking density of 390 cm^2^/bird within the same room to maintain uniform environmental conditions. Each bird was individually tagged with a numbered plastic leg ring. For each age group, live body mass was measured using a digital balance (± 0.1 g accuracy) on day 0 (start of carryover effect trial), day 7 (midpoint), and day 14 (at the end of the trial). During the adult phase of the carryover effect trial, egg number and egg mass were recorded daily throughout the trial. At the end of the experiment, at day 14 of each trial, birds were humanely euthanised by cervical dislocation and the whole liver, brain, and ovary tissues were weighed using a digital balance (± 0.01 g accuracy). In addition, blood samples were collected from the jugular vein into EDTA-coated tubes from the sampled birds. Plasma was separated by centrifugation at 3000 × g for 10 min, immediately collected, flash-frozen, transported to the laboratory, and stored at −80 °C until further biochemical analysis. The plasma biochemical analysis was performed by the URIT-3000 Vet, an automated haematology analyser (Urit-3000 VetPlus, Orvos Technika Ltd., Budapest). The analysed biochemical parameters were glucose, triglycerides, and cholesterol expressed in mmol/l, and total protein and albumin, expressed in g/l. These parameters were measured using an endpoint assay. In contrast, Alanine aminotransferase and Aspartate aminotransferase were measured using a kinetic assay and expressed in units per litre (U/L). Kinetic assays were performed in triplicate, whereas endpoint assays were performed in duplicate, and all assays were carried out according to the manufacturer's protocol. The average values were calculated for statistical analysis.

### 2.3 Statistical analysis

All statistical analyses were performed using R v. 4.2.2 (R Core Team, 2024). Data visualisation was conducted using the ‘ggplot2’ package (version 3.4.3). During analysis, the experimental unit varied across the response variables. Individual birds were considered as experimental units for body mass change, average daily body mass gain, organ mass, and plasma biochemical analysis. In contrast, the cage was considered an experimental unit for feed intake, FCR, and egg production analysis, as the data were collected at the cage level. To evaluate whether the treatment, time period and age had a persistent effect on body mass change across the 14-day trial period, we fitted linear mixed models (LMMs) using the ‘lmer’ function from the ‘lme4’ package (Bates et al., 2015), with birds' identity included as a random factor. Body mass change was calculated as the difference in body mass between consecutive weeks. The significance of fixed effects was assessed using the ‘lmerTest’ package (version 3.1.3) (Kuznetsova et al., 2017). Since the relationship between organ and body mass using log–log regression is not isometric, we analysed organ mass using body mass as a covariate (ANCOVA). Organ mass, average daily body mass gain, feed intake, and FCR were analysed using a linear model, with treatment and age considered as fixed factors. FCR was calculated as total feed intake divided by total body mass gain. Average egg number and egg mass were analysed using a linear model, with treatment considered as a fixed factor. Linear models were fitted for each biochemical parameter analysis, with treatment and age as fixed effects. Pairwise comparisons were tested using the Tukey honestly significant difference (HSD) test, which controls multiple comparisons.

## 3 Results

### 3.1 Carryover effect of treatments on body mass change and gain

**Fig. 1:**
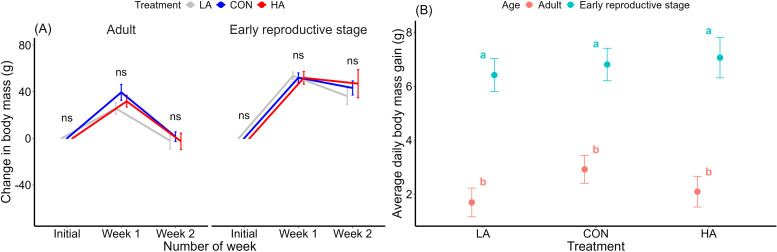
Dietary arginine carryover effect on body mass change and gain in the early reproductive stage and adult phase of female Japanese quail. A) change in body mass across the carryover period, analysed using a linear mixed-effects model. B) average daily body mass gain analysed using a linear model. Data are means ± SEM. ns, not significant at p > 0.05. Different letters indicate significant differences among age groups at p < 0.05. LA, low arginine; CON, control; HA, high arginine.

Dietary arginine showed no clear carryover effect on body mass change and on daily average body mass gain in the early reproductive stage and adult phase of female Japanese quail (treatment: F_2,__261_ = 0.95, p = 0.38, Fig. 1A; F_2,__87_ = 1.04, p = 0.35, Fig. 1B, respectively). In contrast, body mass change and daily average body mass gain were significantly affected over time across different age groups (week × age: F_2,__261_ = 26.22, p < 0.001, Fig. 1A, age: F_1,__87_ = 83.77, p < 0.001, Fig. 1B respectively), with adult birds showing higher body mass loss and early reproductive stage birds showing higher average daily body mass gain during the trial period.

### 3.2 Effect of dietary arginine on organ mass

**Fig. 2:**
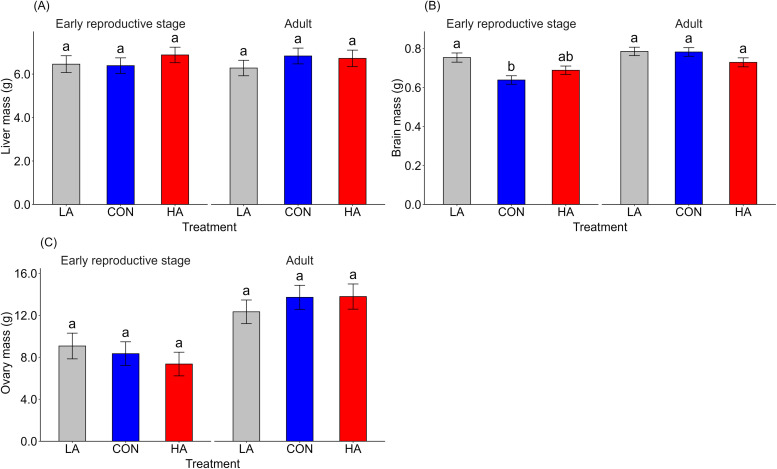
Organ mass across age groups and treatments. A) liver mass. B) brain mass. C) ovary mass. Data are analysed using analysis of covariance (ANCOVA), with body mass included as a covariate and presented as means ± SEM. Different letters indicate significant differences among treatments, p < 0.05. LA, low-arginine; CON, control; HA, high-arginine.

Post-treatment and age interaction showed a significant effect on brain mass (F_2,__41_ = 4.25, p = 0.02, Fig. 2B). Birds previously fed low arginine showed a significantly higher brain mass at the end of the carryover period, in the early reproductive stage female Japanese quail. In contrast, ovary mass differed significantly between age groups (F_1, 41_= 67.86, p < 0.001), with adult birds exhibiting higher relative ovary mass than early reproductive stage birds.

### 3.3 Effect of dietary arginine on egg production

**Fig. 3:**
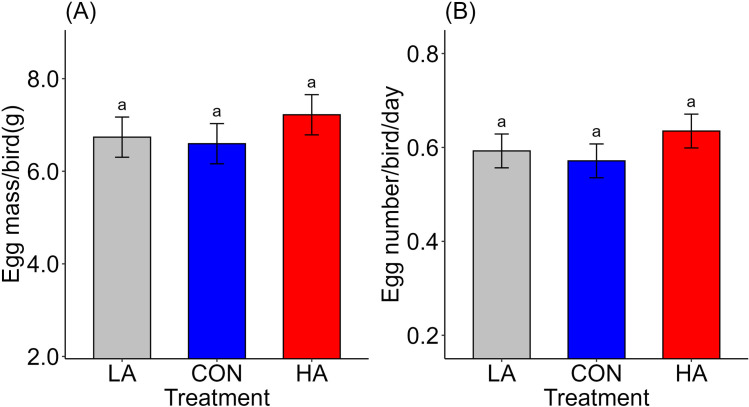
Carryover effect of dietary arginine on egg production. A) egg mass per bird. B) egg number per bird per day. Data are means ± SEM. Similar letters indicate there are no significant differences at p > 0.05. LA, low arginine; CON, control; HA, high arginine.

Dietary arginine did not have a lasting effect on egg mass (F_2,__6_ = 0.56; p = 0.59) and egg number (F_2,__6_ = 0.80; p = 0.48) in adult-phase Japanese quail during the carryover period (Fig. 3A & B).

### 3.4 Effect of treatment residual on daily feed intake and FCR

**Fig. 4:**
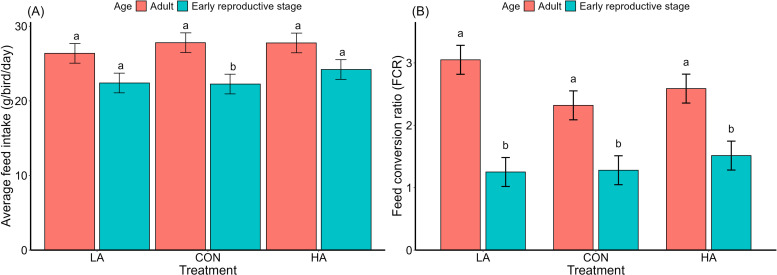
The carryover effect of dietary arginine on feed intake and FCR in the early reproductive stage and the adult phase of birds. A) average feed intake per bird per day. B) feed conversion ratio. Data are means ± SEM. Different letters indicate there are significant differences between age groups at p < 0.05. LA, low arginine; CON, control; HA, high arginine.

During the carryover trial, treatment had no effect (F_2,__12_ = 0.74, p = 0.49, Fig. 4A) on average daily feed intake across the treatments. However, the age of birds showed a significant effect (F_1,__12_ = 16.36, p < 0.01) on average daily feed intake during the trial period. Additionally, FCR was influenced by the age of birds (F_1,__12_ = 47.44, p < 0.001, Fig. 4B); however, treatment had no significant residual effect (F_2,__12_ = 1.21, p = 0.33) on it.

### 3.5 Persistent effect of dietary arginine on blood biochemical parameters

The persistent effect of arginine supplementation showed reduced glucose levels in adult-phase birds (p < 0.001; Table 4). Adult birds also exhibited lower cholesterol concentrations than early reproductive stage birds (age: F_1,__42_ = 23.96; p < 0.001; Table 3). Similarly, adult birds previously subjected to arginine restriction showed decreased ALT/GPT levels (treatment: F_2,__42_ = 4.91, p < 0.01; age: F_1,__42_ = 19.88, p < 0.001; Table 3 & 4), and higher AST/GOT levels (treatment: F_2,__42_ = 4.47, p < 0.01; age: F_1,__42_ = 44.65, p < 0.001; Table 3 & 4).

**Table 3:** Pairwise comparison of age effects on biochemical parameters in each treatment group.

Parameter	Contrast	trt	Estimate	SE	*df*	t-ratio	P-value
ALB (g/l)	Adult–Early reproductive stage	LA	−7.46	4.29	42	−1.74	0.090
	Adult–Early reproductive stage	CON	−8.20	4.29	42	−1.91	0.060
	Adult–Early reproductive stage	HA	−5.57	4.29	42	−1.3	0.200
CHOL (mmol/l)	Adult–Early reproductive stage	LA	−6.36	2.14	42	−2.98	0.005
	Adult–Early reproductive stage	CON	−4.22	2.14	42	−1.98	0.050
	Adult–Early reproductive stage	HA	−7.53	2.14	42	−3.53	0.001
GLU (mmol/l)	Adult–Early reproductive stage	LA	5.55	4.67	42	1.18	0.241
	Adult–Early reproductive stage	CON	11.68	4.67	42	2.5	0.016
	Adult–Early reproductive stage	HA	−6.86	4.67	42	−1.47	0.149
TP (g/l)	Adult–Early reproductive stage	LA	11.52	24.1	42	0.47	0.635
	Adult–Early reproductive stage	CON	8.22	24.1	42	0.34	0.734
	Adult–Early reproductive stage	HA	−3.65	24.1	42	−0.15	0.880
TRI (mmol/l)	Adult–Early reproductive stage	LA	−0.57	3.69	42	−0.15	0.878
	Adult–Early reproductive stage	CON	2.43	3.69	42	0.66	0.512
	Adult–Early reproductive stage	HA	4.36	3.69	42	1.18	0.242
AST/GOT (U/L)	Adult–Early reproductive stage	LA	−53.70	29.5	42	−1.82	0.075
	Adult–Early reproductive stage	CON	−135.00	29.5	42	−4.57	0.001
	Adult–Early reproductive stage	HA	−153.00	29.5	42	−5.19	0.001
ALT/GPT (U/L)	Adult–Early reproductive stage	LA	−41.13	10.1	42	−4.07	0.001
	Adult–Early reproductive stage	CON	−29.49	10.1	42	−2.92	0.006
	Adult–Early reproductive stage	HA	−7.49	10.1	42	−0.74	0.463

Age effects on biochemical parameters in each treatment group. TRI: triglycerides, TP: total protein, GLU: glucose, CHOL: cholesterol, ALB: albumin, ALT/GPT: Alanine aminotransferase, AST/GOT: Aspartate aminotransferase. Data are Tukey-adjusted pairwise comparisons. CON: control, HA: high arginine, LA: low arginine. trt: treatment, SE: standard error, df: degrees of freedom.

**Table 4:** Pairwise comparison of treatment effect on biochemical parameters within each age group.

Parameter	Contrast	Age	Estimate	SE	*df*	t-ratio	P-value
ALB (g/l)	LA–CON	Adult	−1.73	4.29	42	−0.40	0.914
	LA–HA	Adult	−1.78	4.29	42	−0.41	0.909
	CON–HA	Adult	−0.05	4.29	42	−0.01	0.999
	LA–CON	Early reproductive stage	−2.47	4.29	42	−0.57	0.833
	LA–HA	Early reproductive stage	0.10	4.29	42	0.02	0.999
	CON–HA	Early reproductive stage	2.57	4.29	42	0.59	0.820
CHOL (mmol/l)	LA–CON	Adult	−0.23	2.13	42	−0.10	0.993
	LA–HA	Adult	0.53	2.13	42	0.24	0.966
	CON–HA	Adult	0.76	2.13	42	0.35	0.932
	LA–CON	Early reproductive stage	1.90	2.13	42	0.88	0.649
	LA–HA	Early reproductive stage	−0.64	2.13	42	−0.30	0.951
	CON–HA	Early reproductive stage	−2.54	2.13	42	−1.19	0.465
GLU (mmol/l)	LA–CON	Adult	−9.83	4.67	42	−2.10	0.101
	LA–HA	Adult	10.43	4.67	42	2.23	0.077
	CON–HA	Adult	20.26	4.67	42	4.33	0.001
	LA–CON	Early reproductive stage	−3.70	4.67	42	−0.79	0.710
	LA–HA	Early reproductive stage	−1.97	4.67	42	−0.42	0.906
	CON–HA	Early reproductive stage	1.72	4.67	42	0.36	0.927
TP (g/l)	LA–CON	Adult	−22.58	24.1	42	−0.93	0.620
	LA–HA	Adult	−0.42	24.1	42	−0.01	0.999
	CON–HA	Adult	22.16	24.1	42	0.92	0.631
	LA–CON	Early reproductive stage	−25.88	24.1	42	−1.07	0.535
	LA–HA	Early reproductive stage	−15.59	24.1	42	−0.64	0.795
	CON–HA	Early reproductive stage	10.28	24.1	42	0.42	0.904
TRI (mmol/l)	LA–CON	Adult	−2.65	3.68	42	−0.72	0.752
	LA–HA	Adult	−3.91	3.68	42	−1.06	0.542
	CON–HA	Adult	−1.26	3.68	42	−0.34	0.937
	LA–CON	Early reproductive stage	0.35	3.68	42	0.09	0.995
	LA–HA	Early reproductive stage	1.01	3.68	42	0.276	0.958
	CON–HA	Early reproductive stage	0.66	3.68	42	0.18	0.982
AST/GOT (U/L)	LA–CON	Adult	74.90	29.49	42	2.53	0.038
	LA–HA	Adult	111.92	29.49	42	3.79	0.001
	CON–HA	Adult	37.01	29.49	42	1.25	0.428
	LA–CON	Early reproductive stage	−6.02	29.49	42	−0.20	0.977
	LA–HA	Early reproductive stage	12.65	29.49	42	0.42	0.903
	CON–HA	Early reproductive stage	18.68	29.49	42	0.63	0.802
ALT/GPT (U/L)	LA–CON	Adult	−14.96	10.11	42	−1.48	0.310
	LA–HA	Adult	−39.12	10.11	42	−3.86	0.001
	CON–HA	Adult	−24.15	10.11	42	−2.38	0.054
	LA–CON	Early reproductive stage	−3.32	10.11	42	−0.32	0.942
	LA–HA	Early reproductive stage	−5.47	10.11	42	−0.54	0.851
	CON–HA	Early reproductive stage	−2.15	10.11	42	−0.21	0.975

Treatment effect on biochemical parameters within each age group. TRI: triglycerides, TP: total protein, GLU: glucose, CHOL: cholesterol, ALB: albumin, ALT/GPT: Alanine aminotransferase, AST/GOT: Aspartate aminotransferase. Data are Tukey-adjusted pairwise comparisons. CON: control, HA: high arginine, LA: low arginine. trt: treatment, SE: standard error, df: degrees of freedom.

## 4 Discussion

Growth trajectories and growth rates play an important role in feeding and breeding management in avian species (Narinc et al., 2010). Dietary arginine is an essential amino acid for birds that can regulate growth. Therefore, early-life exposure to arginine is expected to affect later-life physiological responses. We previously showed that dietary arginine restriction reduced body mass in immature Japanese quail, whereas arginine supplementation increased egg production in adults (Gashew et al., 2025, 2026a). In contrast, arginine supplementation did not have a significant effect on the growth performance traits of quail (Kheiri & Landy, 2020). However, the current study found that arginine did not have a long-term effect after the birds resumed a recommended diet. This may be because birds showed metabolic recovery and compensatory growth while they resumed a standard diet. Although the carryover effect of dietary arginine did not significantly affect body mass changes and average daily body mass gain in either early reproductive stage or adult quails, adult birds exhibited a higher body mass loss, and early reproductive stage birds gain more average daily body mass (Fig. 1). Together, these findings suggest that body mass change and average daily body mass gain are age-dependent, and the absence of a significant carryover effect indicates that dietary arginine does not exert a long-term influence on body mass in either age group. This may imply that the age of birds influences their growth rate, regardless of the carryover effect of previous dietary treatment effects.

Organ development reflects the overall condition of poultry, which includes nutrition (Richardson et al., 2025). There are conflicting reports on the effects of dietary arginine on organ mass in quails. Some studies have shown that different levels of arginine supplementation increase organ weights in Japanese quail (Al-Daraji et al., 2012), whereas others have reported no significant effects (Al-Tamimy et al., 2025). In our previous report, low dietary arginine was associated with an immediate significant reduction in brain mass in grower quails (Gashew et al., 2026b). In the current study, birds previously fed low dietary arginine had significantly higher brain mass in the early reproductive stage (Fig. 2B). This response may reflect tissue-specific effects of arginine, as previously suggested (Ali et al., 2025). In contrast, brain and liver masses were comparable between the early reproductive stage and adult phase quails, showing that the sizes of these organs are maintained across different age stages regardless of dietary arginine carryover effects. However, a clear difference was recorded in ovary mass, with adult quails exhibiting significantly higher ovary mass than early reproductive stage birds, which is expected because of sexual maturation (Gupta, 2018).

We have previously reported that dietary arginine had an immediate influence on egg number but not on egg mass (Gashew et al., 2026a). However, in the current study, no significant carryover effects of dietary arginine were observed on either egg mass or egg number. This may be because dietary arginine affects reproductive activity only during the supplementation period. This result is consistent with a previous report that the birds' prior dietary conditions do not affect the egg-laying rate (Vedder & Beccardi, 2025). Therefore, either restriction or supplementation of arginine is unlikely to have a long-term effect on egg production once birds are restarted on nutritionally adequate diets.

Feed intake and FCR are indicators of feed consumption and growth performance, and they can be affected by several factors, including the composition of the feed and the age of birds. Feed conversion is a complex biological process and is influenced by numerous factors (Varkoohi et al., 2011). Lower FCR values reflect greater efficiency in converting feed into body mass gain and are associated with higher body weight gain (Varkoohi et al., 2010). Dietary arginine has been reported to have immediate effects on feed intake and FCR in quail. Arginine supplementation has been reported to improve feed intake and FCR (Al-Daraji et al., 2012; Kalvandi et al., 2022), whereas arginine restriction reduces feed intake (de Lima et al., 2022; Sousa et al., 2022). There is limited research on the carryover effect of dietary arginine on feed intake and FCR. In this study, the lower FCR observed in birds at the early reproductive stage reflects their higher body mass gain compared with birds in the adult phase. Furthermore, the absence of significant carryover effects of treatment on feed intake and FCR within the same age group may be attributed to the lack of persistent effects on growth, suggesting that returning to a nutritionally adequate diet restored normal feed intake and FCR.

Blood chemistry is an indicator of health and welfare in poultry (Rajman et al., 2006). Dietary modification is known to affect blood biochemical responses in quail, including glucose, triglycerides, and protein metabolism (Atakisi et al., 2009; Eberhart et al., 2021; Gümüş et al., 2023). Previous studies on arginine supplementation have reported variable effects on plasma metabolites, with some showing reductions in triglyceride and cholesterol concentrations and others showing changes in glucose and total protein levels (Al-Daraji et al., 2012; Kheiri & Landy, 2020). These inconsistencies suggest that the metabolic response to arginine may depend on the physiological stage and the level of nutritional conditions.

During the nutritional treatment period, arginine insufficiency has been linked to increased plasma ALT/GPT levels, and adequate dietary arginine availability helps stabilise hepatic amino acid metabolism (Uyanga et al., 2023). On the other hand, it has been noted that high intake of arginine raises serum AST/GOT levels (Ali et al., 2025). In the present study, unlike body mass changes, average daily body mass gain, FCR, and egg production parameters, dietary arginine showed a significant post-treatment effect on selected plasma biochemical parameters such as glucose, ALT/GPT, and AST/GOT concentrations in adult quail. It demonstrates that some metabolic activities are still influenced by previous nutritional interventions. This could be due to the role of dietary arginine in regulating hormonal processes that affect glucose, fatty acid, and amino acid metabolism (Flynn et al., 2002). The lower glucose concentrations found in adult birds previously fed high arginine may be related to arginine’s role in stimulating insulin secretion, which enhances peripheral glucose uptake (Colca & Hazelwood, 1982; Hassan et al., 2021). Age and treatment-related discrepancies were also revealed in hepatic enzymes, and it may be that age has a greater effect on blood biochemistry (Agusti Montolio et al., 2018; Livingston et al., 2020; Orakpoghenor et al., 2021). Additionally, it has been reported that physiological parameters, including blood glucose concentrations, change as birds transition from one developmental stage to another (Vatsalya & Arora, 2011). Reduced ALT/GPT levels in adults from the low-arginine group after dietary withdrawal may reveal altered amino acid metabolism or hepatic activity during the recovery period. Similarly, changes in AST/GOT concentrations suggest that early dietary arginine levels may have transient effects on liver-associated metabolic processes. These findings highlight that early dietary arginine manipulation induces limited but noticeable age-dependent changes in selected biochemical parameters after withdrawal, while broader metabolic alterations were not detected. Overall, these findings advance our understanding of nutritional programming in Japanese quail and provide a basis for future research into the molecular mechanisms underlying the carryover effects of dietary arginine.

## 5 Conclusion

The present study showed that the carryover effects of dietary arginine were generally limited in Japanese quail after the birds resumed a normal diet. For most productive performance traits, such as body mass change, average daily body mass gain, feed intake, FCR, and egg production, dietary arginine had no persistent effect. However, during the early reproductive stage, previous low-dietary arginine intervention had a significant effect only for brain mass. On the other hand, age significantly influenced body mass change, average daily body mass gain and FCR, with early reproductive stage birds showing higher body mass gain and better FCR than those in the adult phase. In contrast, selected plasma biochemical parameters were influenced by previous arginine levels, with high arginine carryover associated with lower glucose levels and lower arginine associated with higher AST/GOT and reduced ALT/GPT levels in adult birds. In general, age had a stronger influence on production performance traits and selected plasma biochemical profiles than dietary treatment history. These findings suggest that the effects of early-life dietary arginine intervention are largely limited after birds resume a nutritionally adequate diet, although some metabolic effects may persist. Overall, this study provides insights into the carryover effect of early dietary arginine intervention and its effect on later-life performance in Japanese quail.

## Ethics declaration

Animal subject

This study was conducted in accordance with the ARRIVE (Animal Research: Reporting of In Vivo Experiments) guidelines.

This study was approved by the University of Debrecen.

(Approval No 5/2021/DEMÁB)

## Funding

The study was supported by the National Development, Research and Innovation Office, Hungary (K139021 & ADVANCED 153291). M.G. is supported by a Stipendium Hungaricum Scholarship from Tempus Public Foundation for PhD studies.

## CRediT authorship contribution statement

**Mequanint Gashew:** Writing – review & editing, Writing – original draft, Visualization, Validation, Software, Methodology, Investigation, Formal analysis, Data curation, Conceptualization. **Gebrehaweria K. Reda:** Writing – review & editing. **Renáta Knop:** Writing – review & editing. **Csaba Szabó:** Writing – review & editing, Methodology. **Ádám Z. Lendvai:** Writing – review & editing, Supervision, Project administration, Investigation, Data curation, Conceptualization. **Levente Czeglédi:** Writing – review & editing, Supervision, Project administration, Methodology, Funding acquisition, Data curation, Conceptualization.

## Declaration of competing interest

The authors declare that they have no known competing financial interests or personal relationships that could have appeared to influence the work reported in this paper.

## Data Availability

The raw data supporting the conclusions of this article will be made available by the authors, without undue reservation.
